# A Transcriptomics-Based Machine Learning Model Discriminating Mild Cognitive Impairment and the Prediction of Conversion to Alzheimer’s Disease

**DOI:** 10.3390/cells13221920

**Published:** 2024-11-19

**Authors:** Min-Koo Park, Jinhyun Ahn, Jin-Muk Lim, Minsoo Han, Ji-Won Lee, Jeong-Chan Lee, Sung-Joo Hwang, Keun-Cheol Kim

**Affiliations:** 1 Department of Biological Sciences, College of Natural Sciences, Kangwon National University, Chuncheon 24341, Republic of Korea; mkparc@kangwon.ac.kr; 2 Hugenebio Institute, Bio-Innovation Park, Erom, Inc., Chuncheon 24427, Republic of Korea; jwlee@erom.co.kr (J.-W.L.); dinner89@erom.co.kr (J.-C.L.); 3 Department of Management Information Systems, College of Economics & Commerce, Jeju National University, Jeju 63243, Republic of Korea; jha@jejunu.ac.kr; 4 Precision Medicine Research Institute, Innowl, Co., Ltd., Seoul 08350, Republic of Korea; 5 AI Institute, Alopax-Algo, Co., Ltd., Seoul 06978, Republic of Korea; mshan@alopaxalgo.com; 6 Integrated Medicine Institute, Loving Care Hospital, Seongnam 463400, Republic of Korea; lovingconcern.sjh@gmail.com

**Keywords:** transcriptomics, machine learning, mild cognitive impairment (MCI), Alzheimer’s disease (AD), MCI-to-AD conversion, gene expression, RNA sequencing, biomarkers

## Abstract

The clinical spectrum of Alzheimer’s disease (AD) ranges dynamically from asymptomatic and mild cognitive impairment (MCI) to mild, moderate, or severe AD. Although a few disease-modifying treatments, such as lecanemab and donanemab, have been developed, current therapies can only delay disease progression rather than halt it entirely. Therefore, the early detection of MCI and the identification of MCI patients at high risk of progression to AD remain urgent unmet needs in the super-aged era. This study utilized transcriptomics data from cognitively unimpaired (CU) individuals, MCI, and AD patients in the Alzheimer’s Disease Neuroimaging Initiative (ADNI) cohort and leveraged machine learning models to identify biomarkers that differentiate MCI from CU and also distinguish AD from MCI individuals. Furthermore, Cox proportional hazards analysis was conducted to identify biomarkers predictive of the progression from MCI to AD. Our machine learning models identified a unique set of gene expression profiles capable of achieving an area under the curve (AUC) of 0.98 in distinguishing those with MCI from CU individuals. A subset of these biomarkers was also found to be significantly associated with the risk of progression from MCI to AD. A linear mixed model demonstrated that plasma tau phosphorylated at threonine 181 (pTau181) and neurofilament light chain (NFL) exhibit the prognostic value in predicting cognitive decline longitudinally. These findings underscore the potential of integrating machine learning (ML) with transcriptomic profiling in the early detection and prognostication of AD. This integrated approach could facilitate the development of novel diagnostic tools and therapeutic strategies aimed at delaying or preventing the onset of AD in at-risk individuals. Future studies should focus on validating these biomarkers in larger, independent cohorts and further investigating their roles in AD pathogenesis.

## 1. Introduction

As of 2023, more than 55 million people worldwide are estimated to suffer from Alzheimer’s disease (AD), the most common cause of dementia. This number is projected to increase to 82 million by 2030 and 150 million by 2050, with nearly 10 million new cases each year [1,2,3]. The clinical spectrum of AD encompasses a range from asymptomatic stages to mild cognitive impairment (MCI) and, eventually, to mild, moderate, or severe AD. MCI is characterized by cognitive decline that does not significantly impair an individual’s daily life activities [4]. The risk of progression from MCI to AD dementia increases over time, with approximately 10% of individuals progressing within one year and 80% within six years following an MCI diagnosis [5,6,7]. Identifying MCI and predicting which patients will progress to AD dementia is the most crucial point in initiating timely interventions aimed at slowing or halting disease progression. However, this remains one of the most challenging tasks in the field of AD research.

Primary clinical measures for assessing progression from MCI to AD include neuropsychological tests, such as the Mini-Mental State Examination (MMSE), and neuroimaging biomarkers, such as magnetic resonance imaging (MRI). However, the MMSE is inherently complex and can be somewhat circular, as it diagnoses cognitive impairment based on the severity of decline, which can vary widely in MCI [8]. MRI is limited not only by its high cost but also by the requirement for serial measurements over time to accurately track cognitive changes. Additionally, MRI findings may be confounded by factors unrelated to cognitive decline, such as age-related atrophy [9]. Some plasma biomarkers have demonstrated high diagnostic performance in differentiating MCI and/or AD from cognitively unimpaired (CU) individuals [10,11,12]. Despite substantial research efforts, few studies have conclusively demonstrated biomarkers with the capacity for MCI-specific discrimination, predictive accuracy for MCI-to-AD conversion, or suitability for cognitive decline assessment [13,14,15]. Cognitive changes during the prodromal stage of AD are often subtler than those observed in more advanced stages, making it challenging to longitudinally capture dynamic changes in cognitive and functional abilities. Moreover, the invasive and costly nature of these biomarkers limits accessibility and necessitates specialized settings or equipment. This study proposes that an ML-based approach integrating gene expression data and demographic features can effectively discriminate MCI from CU individuals and predict MCI-to-AD conversion.

Monoclonal antibody drugs targeting amyloid beta (Aβ) bind with high affinity to Aβ fibrils and have been shown to reduce cognitive decline in individuals with MCI or early-stage AD. For instance, lecanemab has been demonstrated to clear Aβ in two-thirds of patients and slow cognitive and functional decline in individuals with MCI or early AD over an 18-month double-blind, placebo-controlled trial [16]. And the anti-Aβ drug donanemab has been reported to slow cognitive decline by up to 60% in individuals who begin treatment in the early stage, such as MCI [17]. Despite their efficacy, these therapies are associated with high costs and significant risks, including amyloid-related imaging abnormalities that can lead to severe side effects, such as a brain hemorrhage and seizures. Therefore, it is imperative to accurately distinguish MCI from CU individuals and identify MCI patients at high risk of progression to AD. This study suggests that RNA and demographic features can effectively identify individuals with early symptoms who are likely to develop AD, thereby optimizing candidate selection for anti-Aβ therapy. This could enable the timely intervention for cognitive decline before the onset of symptomatic AD, potentially reducing the prevalence of AD.

The aim of this study was to investigate whether multimodal data, comprising RNA sequencing, protein immunoassays, demographics, and neuropsychological measures, combined with machine learning (ML) algorithms could effectively discriminate MCI and predict the risk of MCI-to-AD progression. The use of simple, more accessible, and less invasive RNA biomarkers combined with key demographics facilitates this diagnostic approach. While single-modality RNA sequencing is preferred due to cost considerations and the reduced burden of multiple testing, this ML-based approach shows great promise in distinguishing MCI patients from CU individuals, enhancing the therapeutic efficacy of anti-Aβ therapy, and, consequently, reducing the prevalence of AD dementia. Our transcriptomics-based model, which achieved an AUC of 0.97 for discriminating MCI from CU individuals and 0.93 for discriminating AD from MCI in the Alzheimer’s Disease Neuroimaging Initiative (ADNI) cohort, outperformed conventional models that used only neuropsychological scores and demographic data, which yielded AUCs of 0.55 and 0.81, respectively.

## 2. Materials and Methods

### 2.1. Datasets and Participants

All datasets were acquired from the Alzheimer’s Disease Neuroimaging Initiative (ADNI) GO/2 phases (https://ida.loni.usc.edu/, accessed 21 June 2023). The ADNI is regarded as a landmark study in the field of Alzheimer’s disease (AD) research, primarily aimed at evaluating whether neuroimaging and other biological markers can be integrated with clinical and neuropsychological assessments to diagnose and monitor mild cognitive impairment (MCI) and early AD. The diagnosis of Alzheimer’s dementia was determined in accordance with the criteria of the National Institute of Neurological and Communicative Disorders and Stroke and the Alzheimer’s Disease and Related Disorders Association [18]. MCI was diagnosed based on the presence of objective memory impairment that did not meet the criteria for dementia. For this study, 349 participants were categorized into three different AD continuum statuses: cognitively unimpaired (CU), MCI (non-converters), and AD (converters from MCI to AD). The following variables were extracted for analysis: baseline demographics (age, gender, years of education, and ApoE ε4 genotype), baseline neuropsychological test scores, protein immunoassay data, and RNA biomarkers. Descriptive statistics were conducted for each variable, and the associations between variables, as well as the relationship between each variable and outcome, were analyzed using appropriate statistical methods (Table 1 and Table 2). The ADNI study received approval from the institutional review boards of all 57 participating centers, and written informed consent, including permission for analysis and data sharing, was obtained from all participants at the time of enrollment.

### 2.2. Neuropsychological Assessment

Composite scores for executive functioning (ADNI-EF), memory (ADNI-MEM), language (ADNI-Lan), and visuospatial functioning (ADNI-VS) were derived from the ADNI neuropsychological battery using item response theory (IRT) methods [19,20]. Baseline and follow-up data to 48 months were applied to develop each composite score. The ADNI-EF included the category fluency tests for animals and vegetables, the trail-making test (parts A and B), the digit span backwards, the Wechsler adult intelligence scale-revised digit symbol substitution, and five clock drawing items (circle, symbol, numbers, hands, and time). The ADNI-MEM score was constructed using various word lists from the Rey auditory verbal learning test and the Alzheimer’s disease assessment scale-cognitive subscale (ADAS-Cog), the three-word recall items from the MMSE (ball, flag, and tree), and logical memory scores (immediate and delayed) [21]. The ADNI-Lan score was calculated based on a neuropsychological battery that included three language-related tests, six language tasks from the MMSE, three different language tasks from the ADAS-Cog, and three language items from the Montreal cognitive assessment. The ADNI-VS was calculated based on a neuropsychological battery including five tests related to clock copying, the constructional praxis test from the ADAS-Cog, and the copy design test in the MMSE [21]. Multiple validation analyses comparing each composite score to individual tests and global scores were conducted among participants who were CU or had MCI or AD. The MMSE is a 30-item assessment of global cognitive status that covers domains such as orientation, concentration, attention, verbal learning (without delayed recall), naming, and visuoconstruction.

### 2.3. Imputation of Missing Values

Prior to the imputation process, three participants were excluded due to the unavailability of fluorodeoxyglucose positron emission tomography data. Among the remaining 346 participants, 17 missing values from 15 participants were identified, resulting in 1507 available values out of a possible 1524 data points. To address these missing values, a regression-based imputation method was employed using the scikit-learn package in Python, following previously reported procedures [22]. This method utilizes existing data to predict and fill in the missing values. A regressor model was trained on the available feature values (predictors) to predict the target feature with missing values. Once trained, the model was used to estimate the missing values, which were then imputed into the dataset. All default parameters were applied except for “max_iter”, which was set to 100 to limit the number of iterations. This approach leverages the relationships between features to accurately estimate and replace missing values, thereby preserving the dataset’s integrity and completeness for subsequent analyses. By employing this method, we maximized the use of available data, enhancing the reliability of the results derived from the dataset.

### 2.4. Formulation of the Training Dataset

During the screening visit, potential participants were evaluated based on planned assessments, including neuropsychological testing and imaging. Participants who met the inclusion/exclusion criteria proceeded to the baseline assessment, which included additional neuropsychological testing, imaging, and fluid sampling. The Clinical Core of the ADNI recommends making the diagnosis at baseline, as this time point provides the most comprehensive information for an accurate clinical assessment. Clinical assessments may change over time; for example, a participant classified as CU at baseline and month 12 (m12) might be reclassified as MCI at month 24 (m24) and revert to CU at month 36 (m36) and month 48 (m48). In such cases, the participant was allocated to the CU group. Participants were classified as maintaining an MCI diagnosis if they were diagnosed as MCI at baseline and at least three out of five time points.

For the CU group, RNA sequencing data were available for 65 participants. For the MCI group and AD converters, RNA sequencing data were available for 142 and 39 participants, respectively. A total of 207 participants were qualified for training the first classifier (CU vs. MCI), and 181 participants were selected for training the second classifier (MCI vs. AD). All data, including demographics, neuropsychological measures, and transcriptomics, were merged for analysis.

### 2.5. Merging Variables from Different Visits

Analyzing longitudinal data requires appropriate preprocessing to ensure the data’s integrity. The dataset includes variables such as demographics, neuropsychological assessment scores, and RNA expression levels, which were collected at five time points (baseline, m12, m24, m36, and m48). Each variable consists of five data points per participant, which need to be transformed into a single representative value for effective machine learning analysis. Several methodologies were considered for this transformation: “Averaging or median calculation” provides a measure of central tendency, with the median being robust to outliers and skewed distributions. “First observation carried forward” utilizes the baseline value, assuming it is the most representative. “Last observation carried forward” uses the most recent time-point value (m48), assuming it is the most relevant. “Slope of linear regression” fits a regression line to the five time points and uses the slope as the representative value. After comparing these methodologies, the delta averaging ratio was selected. For instance, if a participant’s diagnosis changed from MCI to AD at m24, the delta averaging ratio calculates two proportional increase/decrease ratios: one between baseline and m12 and another between m12 and m24. This approach consolidates multiple time points into a single value and captures the trend or progression of the variable over time, which is particularly relevant for longitudinal studies.

### 2.6. Transcriptomics Data

In the ADNI GO/2 study, whole blood samples from participants were collected in three PAXgene Blood RNA tubes. These tubes were gently mixed via inversion 8–10 times and centrifuged at room temperature within one hour of collection. The centrifugation was conducted at 3000 rpm for 15 min to separate the plasma fraction. Then, the buffy coat was aliquoted into two 2 mL cryogenic vials and shipped to the National Cell Repository for AD within 24 h of collection. Total RNA was purified from whole blood using the PAXgene Blood RNA Kit (Qiagen Inc., Valencia, CA, USA). Detailed protocols are available at the ADNI Methods: https://adni.loni.usc.edu/data-samples/adni-data/biofluid-biomarker/ (accessed on 21 June 2023).

RNA sequencing was performed using the Human Genome U219 Array (Affymetrix, Santa Clara, CA, USA). Raw gene expression data were processed following the standard quality control (QC) procedures established by the ADNI Genetics Core. These procedures included the assessment of RNA quality, overall array assay QC, sex verification, and sample identity checks [23,24]. Raw expression values were preprocessed with the robust multi-chip average normalization method [25]. All probe sets were mapped and annotated to the reference human genome (hg19). Following QC, the RNA expression profiles containing 21,150 probes were adjusted for covariates, including age, gender, and RNA integrity number values.

### 2.7. Machine Learning (ML)

Four ML algorithms, along with linear regression, were employed to identify genes associated with MCI or AD and to differentiate them from those associated with CU or MCI participants: Ridge, Lasso, Support Vector Machine (SVM), and Random Forest. Ridge and Lasso regressions are extensions of linear regression that incorporate regularization techniques to mitigate overfitting. Conventional linear regression can be prone to overfitting, particularly when certain coefficients assume to take on very large values, which can result in poor generalizability to new datasets. Ridge regression uses L2 regularization to penalize the sum of squared coefficients, while Lasso regression applies L1 regularization to penalize the absolute values of the coefficients. Both approaches reduce the likelihood of overfitting by shrinking large coefficients, thereby enhancing the model’s ability to generalize to unseen data. SVM is designed to find an optimal hyperplane that best separates the different classes in the dataset. It can also employ kernel functions to enable non-linear classification, which increases the flexibility and applicability of the model to complex data structures. Random Forest, on the other hand, constructs an ensemble of decision trees, with each tree built on a random subset of features and data. The final classification decision is made by aggregating the outputs from all individual trees, which enhances the robustness and accuracy of the model through a voting mechanism.

The dataset, comprising CU, MCI, and AD participants, was randomly divided into a training set (80%) and a testing set (20%). To assess model robustness, five-fold cross-validation was employed. The ML algorithms were used to build models on the training set, and their performances were evaluated on the testing set. The model with the highest AUC was selected as the best model for distinguishing MCI from CU participants. The best model was further validated using the testing data, with AUC serving as the primary performance metric.

### 2.8. Gene Set Enrichment Analysis (GSEA)

GSEA was performed to identify differentially expressed genes (DEGs) and enriched biological processes across different cognitive states [26,27]. The first dataset included gene expression data from CU and MCI individuals, while the second dataset comprised data from MCI and AD individuals. The GSEA were conducted using version 4.3.3 for Windows, available from the GSEA website (gsea-msigdb.org). The annotated gene set c5.go.bp.v2023.2.Hs.symbols.gmt from the MSigDB was selected as a reference, categorizing biological processes (BPs) by listing the genes involved in each process. The number of permutations was set to 1000 (default), and the permutation type was set to gene_set, rearranging the gene sets of the BPs for statistical testing. Default settings were used for the metrics for ranking genes and the enrichment statistic. The maximum gene set size was capped at 200 to exclude larger sets, and the minimum size was set at 5 to exclude smaller sets, focusing on biologically significant processes. The significance level was set at *p* < 0.05 or <0.01. A chip file created by converting GPL13667-15572 from the gene expression omnibus was used for collapsing probes to gene symbols. All other conditions followed the default settings of the GSEA, and GO analysis was conducted. Gene expression levels and enrichment scores for each pathway, based on expression changes between two groups, were converted into normalized enrichment score values.

### 2.9. GO and Functional Annotation

To further elucidate the biological functions of DEGs, functional enrichment analysis was conducted, focusing on BPs. Additionally, Kyoto Encyclopedia of Genes and Genomes (KEGG) pathway analysis was utilized to identify the pathways involved in biological molecular interactions. GO and KEGG pathway analyses were performed using DAVID 6.8 or analyzed using Cytoscape 3.10.1, which employs the Benjamini–Hochberg *p*-value correction algorithm to identify significantly enriched terms.

The enrichment library used for GO_BP terms was “GO_BiologicalProcess-EBI-UniProt-GOA-ACAP-ARAP_25.05.2022_00h00”. Selection criteria for representative pathways included GO levels three to eight, a minimum of three genes per term, and mapped genes representing at least 4% of the total associated genes. The Kappa score was used to define term–term interactions and associate terms and pathways with functional groups based on shared genes. Ensemble gene IDs were used as gene identifiers for all analyses. False discovery rate and Bonferroni-corrected *p*-values less than 0.05 were considered significant, indicating that genes within a particular BP were significantly enriched in either the MCI or AD clusters, rather than by random chance.

### 2.10. Protein Immunoassays

The quantification of plasma protein biomarkers was performed using single-molecule array (SIMOA) technology, a highly sensitive, paramagnetic microbead-based sandwich enzyme-linked immunosorbent assay. Samples and controls were transferred to 96-well Quanterix^®^ plates and measured in duplicate on an HD-X platform (Quanterix, Billerica, MA, USA) using a 2-step neat assay. This process involved combining target antibody-coated paramagnetic beads with the sample and a biotinylated detector antibody during the same incubation. Target molecules in the sample were captured by the antibody-coated beads and simultaneously bound by the biotinylated detector antibody. These capture antibody-coated beads were conjugated to streptavidin-β-galactosidase, which labeled the captured protein. The substrate solution enabled β-galactosidase to hydrolyze the substrate into a fluorescent product, providing the measurement signal. The protein concentration was then interpolated from a standard curve obtained via four-parameter logistic regression fitting.

Plasma p-Tau181 was analyzed by the clinical neurochemistry laboratory at the University of Gothenburg, following previously reported procedures [10]. Biotinylated anti-pTau181 was used as the capture antibody (AT270, Thermo Fisher Scientific, Waltham, MA, USA), and Tau12 antibody was utilized as the detector (BioLegend, San Diego, CA, USA). Plasma neurofilament light chain (NFL) protein was also analyzed using the NF-light kit on the SIMOA HD-X analyzer, as previously reported [28]. Plasma samples were measured at 1:4 dilution and in a blinded manner. The analytical lower limits of quantification were 0.4 pg/mL for NFL and 1.0 pg/mL for p-tau181. The mean intra-assay coefficient of variation was less than 5%.

### 2.11. Cox Proportional Hazards Analysis

To identify the genes associated with the conversion of MCI to AD dementia, Cox proportional hazards analysis was performed on the 96 genes selected through GSEA. This analysis evaluates the association between multiple independent variables and survival rates, considering RNA expression levels as a treatment variable due to the absence of treatment data. Each gene was mapped to one or more probes using the chip platform, and probe expression values were converted into tertiles (low, intermediate, or high). Tertile cut-offs were determined independently for each probe. The analysis was conducted for 353 models (one for each probe). Demographic information (age, sex, years of education, and ApoE ε4 status) was also included, resulting in 2 × 353 models (with and without covariates).

### 2.12. Sensitivity Analysis for SVM Model Outcomes

To validate the significance of the identified RNA biomarkers, a generalized regression model was employed, chosen for its flexibility in handling high-dimensional data. From the comparison of CU and MCI participants, four types of ML techniques identified 353 RNA biomarkers with statistically significant associations (Bonferroni-corrected *p* < 0.05). Elastic net regression and the corrected Akaike information criterion (AICc) were used for estimation and validation, respectively. The statistical significance of RNA biomarkers was evaluated through *p*-values obtained from the regression coefficients in the generalized regression model. Key performance metrics (precision, accuracy, and F1 score) were compared with those of the SVM model. A correlation heatmap was plotted using JMP Pro 17 to visualize the matrix of correlations between parameter estimates.

### 2.13. Linear Mixed-Effects Model (LMM)

To evaluate the predictive power of baseline pTau181 and NFL protein biomarkers in relation to cognitive decline over a four-year follow-up period, an LMM was employed, as detailed previously [29]. The LMM incorporated both fixed and random effects. Fixed effects included baseline plasma protein biomarker levels (categorized into tertiles), time (years from baseline), and the interaction between biomarker levels and time. Random intercepts accounted for inter-individual variability in baseline cognitive performance, while random slopes for time-captured individual differences in cognitive trajectories. The dependent variable was the neuropsychological measure (MMSE, ADNI-MEM, -EF, -LAN, or -VS), with the plasma biomarker levels, time, and their interaction as predictors. The covariates included the age, sex, years of education, and ApoE ε4 status.

### 2.14. Statistical Analysis

Demographic differences among groups were assessed using analysis of variance with Tukey’s multiple comparisons test for continuous variables and Pearson’s Chi-squared test for categorical variables. In the sensitivity analysis of SVM model outcomes, a binomial generalized regression analysis was conducted using JMP Pro 17 to identify significant RNA biomarkers for discriminating MCI from CU individuals. Cox proportional hazards analysis was employed to evaluate the associations between these biomarkers and the risk of conversion from MCI to AD dementia. All statistical analyses were performed using R software, version 4.3.3 (R Foundation for Statistical Computing), JMP Pro 17 (SAS Institute Inc., Cary, NC, USA), IBM SPSS version 27.0 (IBM, Inc., New York, NY, USA), and MedCalc version 22.009 (MedCalc software, Belgium). Statistical significance was set at a two-tailed *p*-value of less than 0.05.

## 3. Results

**Figure 1:**
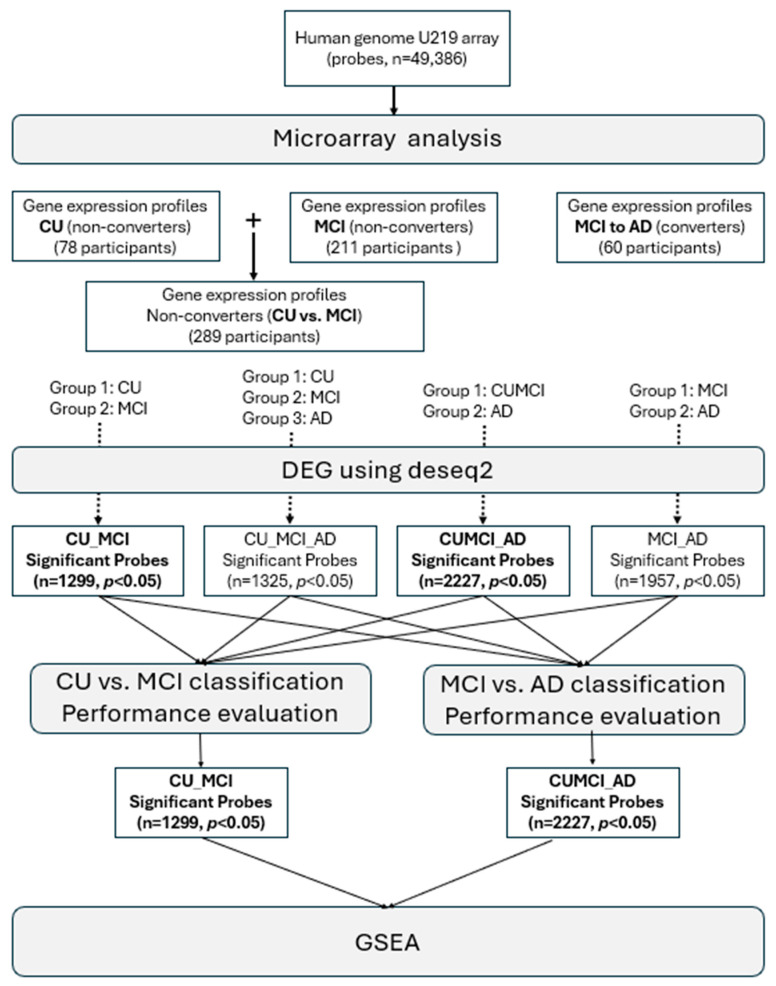
Study design and workflow for identifying RNA biomarkers predictive of MCI or AD. Methods for biomarker identification and functional annotation are depicted in grey round squares. The numbers in parentheses at the bottom indicate the RNA probes that met the selection criteria and were subsequently identified using ML algorithms. Abbreviations: CU (cognitively unimpaired), MCI (mild cognitive impairment), AD (Alzheimer’s disease), DEG (differentially expressed gene), and GSEA (gene set enrichment analysis).

The study design and workflow for identifying RNA biomarkers predictive of MCI or AD are summarized in a flowchart, which illustrates the methods and procedures utilized in this research (Figure 1).

### 3.1. Baseline Characteristics

Baseline characteristics were analyzed by stratifying the participants into three groups: CU, MCI (non-converters), and MCI (converters to AD). Age was found to be significantly different among these groups (*p* = 0.007, Table 1); however, when stratified by conversion status, age did not display a significant difference (Table 2). ApoE ε4 status and neuropsychological measures, including MMSE, ADNI-MEM, ADNI-EF, ADNI-LAN, and ADNI-VS, exhibited statistically significant differences across both diagnosis and conversion stratifications (*p* < 0.01), as detailed in Table 1 and Table 2. In contrast, gender and years of education did not present significant differences in either stratification. An analysis using a scatter plot matrix did not reveal significant associations between demographic variables (age, sex, ApoE ε4 status, and years of education) and MCI classification, suggesting that these variables alone are inadequate for reliably classifying disease status (Figure S1a).

We further investigated the predictive utility of neuropsychological measures for classifying disease status (Figure S1b). The correlation matrix for various combinations of baseline neuropsychological scores indicated that while many red dots were concentrated on the left and lower sides of some plots—indicating potential associations—red dots were also scattered across other regions of the plots. This distribution suggests that neuropsychological measures alone may not be sufficient to reliably predict conversion from MCI to AD.

### 3.2. Transcriptomics-Based RNA Biomarkers and Accuracy Comparisons

The participants were stratified along the AD continuum into three groups: CU, MCI, and AD. The baseline gene expression profiling dataset included 735 participants and 49,386 RNA probes. To identify DEGs relevant for discriminating MCI or AD, we employed DESeq2, a comprehensive tool for the gene-level analysis of RNA sequencing data, as previously described [30]. Four AD continuum status combinations were examined: CU vs. MCI (CU_MCI), MCI vs. AD (MCI_AD), CU vs. MCI vs. AD (CU_MCI_AD), and a combined CU and MCI vs. AD (CUMCI_AD), using *p*-value thresholds of <0.01 and <0.05. The CU_MCI combination indicates RNA probes that can significantly differentiate MCI from CU patients.

To evaluate the classification performance of the RNA probe sets, we employed four ML algorithms: Ridge, Lasso, SVM, and Random Forest (RF) (Table 3). Five-fold cross-validation was performed, and the mean accuracy across the five tests was calculated. As detailed in Table 3, for the CU vs. MCI comparison, the CU_MCI and CU_MCI_AD combinations achieved accuracies of 0.83 or higher. In contrast, the CUMCI_AD and MCI_AD combinations exhibited accuracies of 0.81 or less. Conversely, for the MCI vs. AD comparison, the CUMCI_AD and MCI_AD combinations outperformed the CU_MCI and CU_MCI_AD combinations. The mean accuracy for predicting AD presence compared to MCI (MCI vs. AD) was around 0.6, suggesting that gene expression profiling alone does not provide sufficiently robust performance for distinguishing AD from MCI. This finding underscores the need for incorporating additional variables, such as neuropsychological measures or demographic data, to enhance classification accuracy.

### 3.3. Discriminating Performances of Multivariate Models

While gene expression array data demonstrate potential in distinguishing MCI from CU individuals, its utility in discriminating AD from MCI remains limited. This limitation underscores the necessity for a multifaceted approach that integrates additional clinical and demographic variables to enhance discriminative performance. To address this, we investigated the discriminatory power for MCI or AD when RNA probes were combined with demographic variables (age, sex, ApoE ε4, and years of education) alone or together with neuropsychological measures. Two multivariate models were developed: one incorporating gene expression array data with demographics alone and the other extending to include neuropsychological measures as well. As outlined in Section 2.5, the delta averaging ratio strategy was applied consistently to integrate variables from different visits.

**Figure 2:**
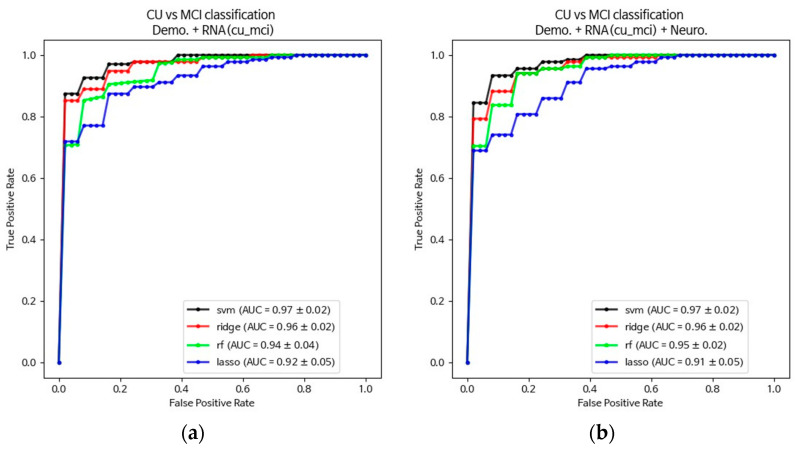

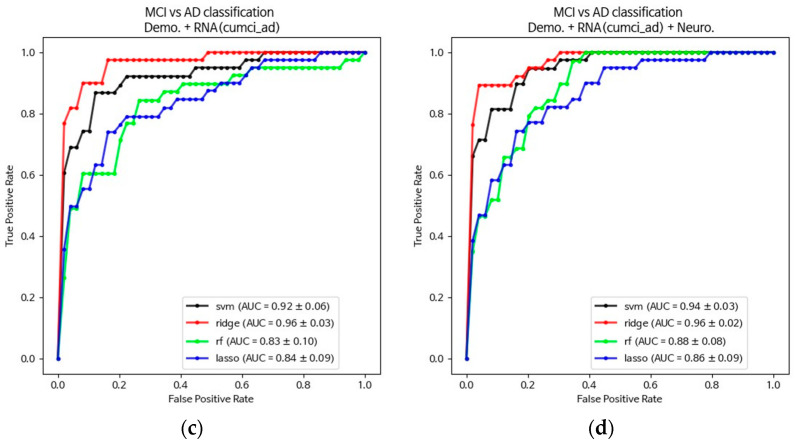
Comparison of the discriminating performances of multivariate models. (**a**) Receiver operating characteristic (ROC) curves for RNA biomarkers combined with demographic variables in the CU vs. MCI comparison. (**b**) ROC curves for RNA biomarkers in combination with demographics and neuropsychological measures in the CU vs. MCI comparison. (**c**) ROC curves for RNA biomarkers combined with demographic variables in the MCI vs. AD comparison. (**d**) ROC curves for RNA biomarkers in combination with demographics and neuropsychological measures in the MCI vs. AD comparison.

The AUC was calculated to assess the overall discriminative capacity of these models. For distinguishing MCI from CU individuals, the SVM and Ridge algorithms consistently showed superior performances across all models, including the basic RNA probe model and multivariate models that incorporated RNA probes, demographic variables, or a combination of RNA probes, demographics, and neuropsychological measures. Among these, the SVM algorithm exhibited the highest AUC (0.97 ± 0.02, Table 3 and Figure 2) across all models at the cut-off *p*-value of 0.05. While the Ridge algorithm performed optimally in the basic RNA probe model, it showed a slight reduction in the AUC for the multivariate models (0.96 ± 0.02, Figure 2). In contrast, the AUC for RF and Lasso algorithms improved in the multivariate models compared to the basic RNA probe model (Table 3; Figure 2). Overall, the SVM model based on the basic RNA probe model is preferred as the parsimonious approach for classifying MCI from CU patients. For differentiating AD from MCI, the multivariate models outperformed the basic RNA probe model, with AUC values for all four ML algorithms falling below 0.84 in the basic RNA probe model. However, when using composite models that integrated RNA probes, demographic variables, and neuropsychological measures, the SVM and Ridge algorithms demonstrated significantly improved discriminative performance. The multivariate models, which included all three variable types, further increased the AUC to 0.94 and 0.96 for the SVM and Ridge algorithms, respectively (Table 3; Figure 2). Thus, for optimal AD classification, the inclusion of demographics and neuropsychological measures was essential. 

From the perspective of algorithms, SVM demonstrated the best performance in discriminating MCI from CU individuals. In contrast, Ridge outperformed the other ML algorithms in distinguishing AD from MCI, although the difference in performance between these two algorithms was negligible. Given the large number of variables involved, collinearity is more likely to occur, and Ridge regression is particularly effective at managing collinearity between variables. Despite the similar discriminative performances of the SVM and Ridge algorithms, the SVM algorithm was preferred due to its superior generalization capacity for binary classification.

### 3.4. GSEA, GO, and Functional Annotation

GSEA was performed under two comparative conditions (CU vs. MCI and MCI vs. AD) to identify relevant gene sets and enriched biological processes across different stages of the AD continuum. Following the procedure and parameters outlined in Section 2.8, the GSEA identified significant gene sets and biological processes (Table 4 and Table 5). Enrichment scores (ES) were calculated to determine whether a gene set was positively or negatively correlated with RNA probe expression levels, while the normalized enrichment score (NES) was used as the standard metric for gene set size variability. GO terms enriched from the comparisons were considered significant with a nominal *p*-value < 0.01.

**Figure 3:**
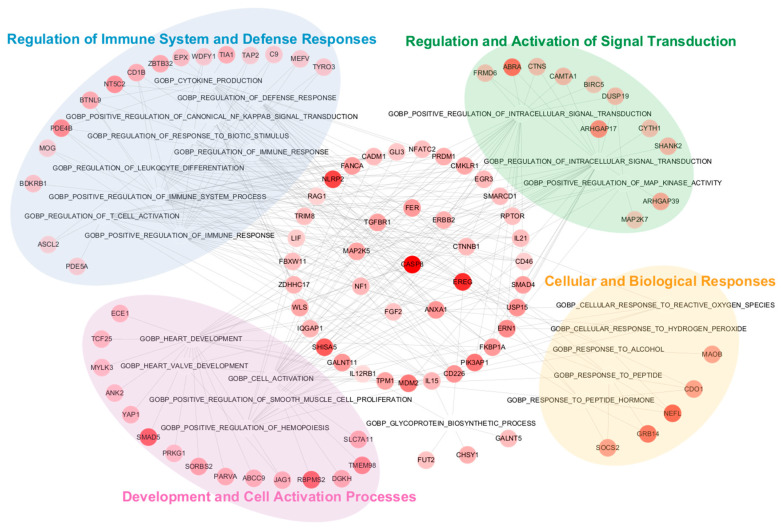
GSEA and gene network analysis for DEGs discriminating MCI from CU individuals. Ten hub genes from upregulated DEGs are displayed in the center small circle, representing those that overlap more than three clusters. Nodes with a deeper red color represent higher rank scores.

To further investigate the role of hub genes in discriminating MCI from CU individuals, as well as AD from MCI, gene network analysis was conducted using Cytoscape. Node and edge data linking GO BP terms to genes were created based on the tsv file information obtained from the GSEA results. In the CU vs. MCI comparison, GO BP terms were grouped into four major ontology clusters based on the hierarchical relationships: (1) regulation of the immune system and defense responses (e.g., “positive regulation of canonical NF-κB signal transduction”), (2) regulation and activation of signal transduction (e.g., “regulation of intracellular signal transduction”), (3) development and cell activation processes (e.g., “positive regulation of hemopoiesis”), and (4) cellular and biological responses (e.g., “cellular response to reactive oxygen species”). Genes associated with two of these clusters were represented by large intermediate circles, while genes such as *FER*, *ERBB2*, *CTNNB1*, *EREG*, *ANXA1*, *FGF2*, *NF1*, *MAP2K5*, and *TGFBR1*, which are involved in three clusters, were depicted as smaller circles within the large ones. The gene *CASP8*, which is involved in all four clusters, was positioned centrally in the network (Figure 3). Representative GO BP terms negatively associated with RNA probes in the CU vs. MCI comparison included those involved in DNA repair, fat cell differentiation, and response to insulin (Table 4).

**Figure 4:**
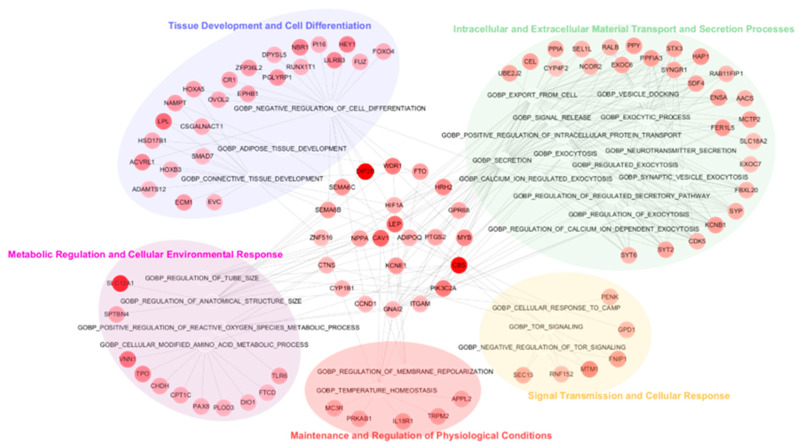
GSEA and gene network analysis for DEGs discriminating AD from MCI. Ten hub genes from upregulated DEGs are displayed in the center small circle, representing those that overlap more than three clusters.

The GSEA results from the MCI vs. AD comparison, along with the top 20 GO BP terms, are presented in Table 5. The GO BP terms identified in this comparison were grouped into five major clusters based on the GO BP hierarchy: (1) intracellular and extracellular material transport and secretion processes (e.g., “regulated exocytosis”), (2) metabolic regulation and cellular environmental response (e.g., “cellular modified amino acid metabolic process”), (3) signal transmission and cellular response (e.g., “TOR signaling”), (4) tissue development and cell differentiation (e.g., “adipose tissue development”), and (5) maintenance and regulation of physiological conditions (e.g., “regulation of membrane repolarization”). Genes involved in two of these clusters were represented by large intermediate circles, while genes such as *HIF1A*, *NPPA*, *PTGS2*, and *KCNE1*, which are associated with three clusters, were depicted as smaller circles within the larger ones. Genes such as *LEP*, *CAV1*, and *APIPOQ*, which were associated with more than four clusters, were positioned at the center (Figure 4). Representative GO BP terms negatively associated with RNA probes in the MCI vs. AD comparison included processes related to DNA repair and damage response, organelle fission, the negative regulation of cell adhesion, and microtubule cytoskeleton organization (Table 5).

### 3.5. Sensitivity Analysis

**Figure 5:**
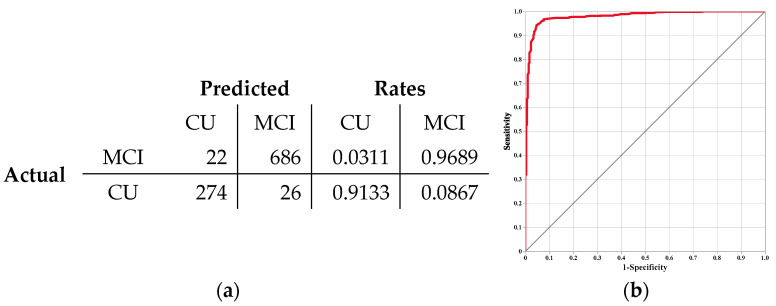
Sensitivity analysis result. (**a**) Confusion matrix for the generalized regression; the sensitivity analysis misclassified 26 cases as CU and 22 cases as MCI out of the 712 MCI and 296 CU cases, respectively. (**b**) ROC curve of the sensitivity analysis, with an AUC of 0.9801.

To identify the RNA biomarkers capable of effectively discriminating MCI from CU individuals, four ML algorithms were initially employed. Among these algorithms, the SVM model demonstrated the highest efficacy, enabling the identification of a robust panel of 353 RNA probes from an initial set of 21,150 probes. To further evaluate the performance of the RNA probes selected by SVM, a generalized regression analysis was conducted using a subset of 100 RNA probes as part of a sensitivity analysis. The elastic net was employed for estimation, and the AICc was used for validation. The discriminative performance metrics achieved were as follows: AUC, 0.9801; sensitivity, 0.9689; specificity, 0.9133; precision, 0.9635; accuracy, 0.9524; F1 score, 0.9662; and Matthew’s correlation coefficient, 0.8857 (Figure 5). These results highlight the potential of the identified RNA biomarkers in accurately differentiating MCI from CU individuals, demonstrating high sensitivity, specificity, and overall accuracy. Additionally, we performed multivariate correlation analyses and examined the correlation of estimates. A clustered correlation heatmap confirmed the robustness of the selected RNA biomarkers, indicating that these biomarkers share biological significance and are interconnected (Figure S2).

### 3.6. Prediction of MCI-to-AD Conversion

**Figure 6:**
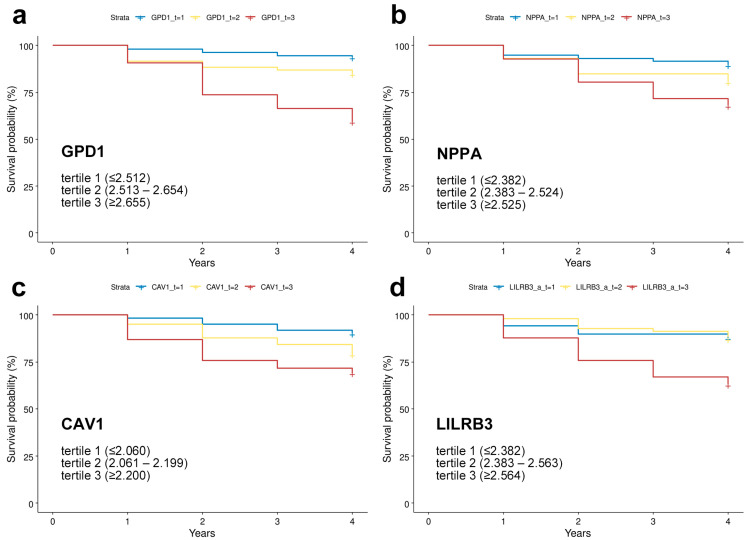
Representative Cox proportional hazard curves for MCI-to-AD converters. Expression values normalized using the robust multi-chip average method per each tertile are indicated in figure insets: (**a**) GPD1; (**b**) NPPA; (**c**) CAV1; (**d**) LILRB3. Year “0” marks the baseline diagnosis. Tick marks represent participants who were AD conversion-free at the last follow-up or who were censored at that time point.

We investigated whether specific hub RNA biomarkers were significantly associated with the conversion from MCI to AD. A total of 271 participants from the ADNI cohort were evaluated for AD conversion over a four-year follow-up period. Of these participants, 60 converted to AD dementia. The initial event was defined as a diagnosis of MCI, with the endpoint being conversion to AD. For those who converted to AD, the survival time was defined as the interval from baseline assessment to AD diagnosis. For non-converters, who were right-censored at the last follow-up, the survival time was set to four years. Cox proportional hazards analysis was conducted using the survival and survminer packages in R software, incorporating various covariates into the models. This analysis revealed that the highest tertile of baseline expression levels of 123 RNA probes was significantly associated with an increased risk of MCI-to-AD conversion (Table 6 and Figure 6; The table listing all 123 genes is available as Supplementary Table S1). Most MCI participants with gene expression levels in the lowest or intermediate tertiles remained stable in their MCI status over the four-year period. In contrast, the participants in the highest tertile exhibited a faster rate of MCI-to-AD conversion (Figure 6).

### 3.7. Longitudinal Cognitive Status Predictors: Plasma pTau181 and NFL

**Figure 7:**
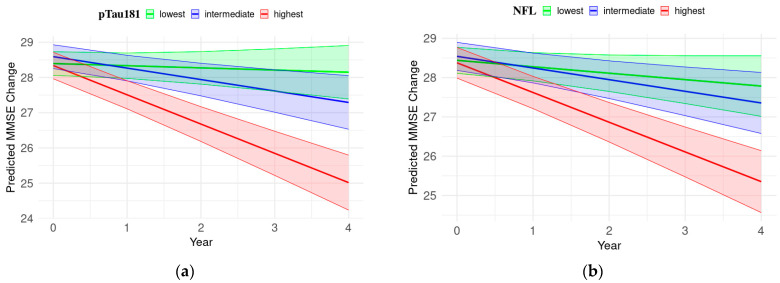

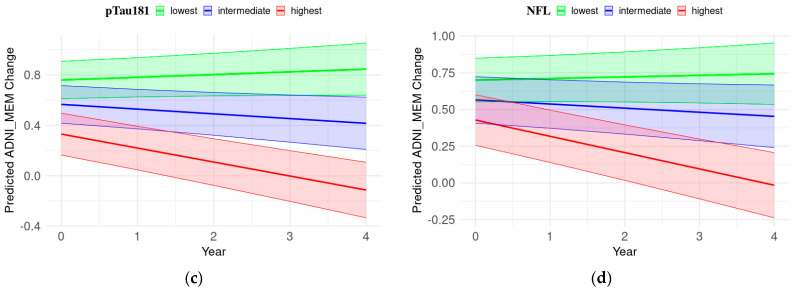
Prediction of longitudinal neuropsychological score alterations based on baseline plasma protein levels. Trajectories were derived from the LMM, with the baseline plasma pTau181 and NFL levels as predictors, being adjusted for age, sex, ApoE ε4, and years of education. MMSE trajectories were stratified by (**a**) pTau181 or (**b**) NFL tertiles, while ADNI-MEM trajectories were stratified by (**c**) pTau181 or (**d**) NFL tertiles. The trajectories depict changes in the MMSE or ADNI-MEM scores over time influenced by different tertiles of baseline pTau181 or NFL levels. The slope, indicative of the rate of cognitive decline, appears steeper for individuals with higher protein levels. The red line represents the highest tertile for each protein, while the blue and green lines represent the intermediate and lowest tertiles, respectively. Shaded areas indicate the 95% confidence intervals of the regression lines. This figure displays the mean levels within each covariate (age and years of education), with females as the reference group. The time span is capped at four years, corresponding to four follow-up assessments.

An LMM analysis was conducted to evaluate the associations between plasma protein biomarkers and cognitive decline, as measured via neuropsychological assessments. To determine the predictive value of plasma biomarkers for cognitive decline, this study compared the baseline concentrations of pTau181 and NFL with the neuropsychological scores. After adjusting for covariates such as age, sex, ApoE ε4 allele, and years of education, the LMM analysis revealed a significant interaction between the highest tertile of pTau181 levels and time across all neuropsychological measures (Table 7; Figure 7). In contrast, the highest tertile of NFL exhibited a significant interaction with time for all cognitive measures except for ADNI-VS. These findings underscore the prognostic value of the plasma pTau181 and NFL levels in predicting cognitive decline. The significant interaction effects with time suggest that individuals with higher baseline levels of these proteins are likely to experience more rapid cognitive deterioration, as evidenced by steeper declines in neuropsychological scores over time (MMSE, ADNI-MEM, -EF, and -LAN). Additional LMM plots are available in Figure S3.

## 4. Discussion

In 2023, the US Food and Drug Administration approved lecanemab, a monoclonal antibody targeting aggregated Aβ peptides, as the first disease-modifying treatment for AD [16]. This drug is indicated for patients with MCI or those in the early stages of AD. Similarly, another anti-Aβ drug, donanemab, was approved in July 2024 [17]. These treatments have demonstrated efficacy in reducing clinical cognitive decline in subjects with early AD during phase 3 clinical trials. However, these monoclonal antibody treatments have several limitations. Anti-Aβ therapies are expensive, with annual costs exceeding USD 26,000, and are cumbersome, requiring biweekly or monthly infusions and regular MRI scans to monitor for severe side effects, such as amyloid-related imaging abnormalities. A critical challenge lies in identifying the patients who will derive the most benefit from these therapies. Differentiating MCI from CU individuals is essential, as these anti-Aβ therapies could provide significant clinical benefits by preventing cognitive decline in patients with early AD. Early diagnosis of AD, prior to the onset of dementia symptoms, is associated with multiple benefits, including prolonged survival, improved psychological well-being for patients and caregivers, and reduced healthcare costs. Recent findings from clinical trials on disease-modifying therapies suggest that achieving meaningful therapeutic success is likely dependent on early intervention [17]. ML and deep-learning algorithms are poised to become essential tools in analyzing and integrating vast datasets in AD research. These technologies can help identify the pathways involved in disease initiation and progression, thereby guiding early diagnosis to mitigate the prevalence of AD. While substantial progress has been made in validating plasma proteins as biomarkers for AD and in predicting cognitive decline in individuals with MCI [10,31,32], this study is, to our knowledge, the first to demonstrate the utility of ML in classifying plasma RNA biomarkers that can discriminate MCI from CU individuals, as well as predict the progression to AD dementia in patients with cognitive decline symptoms. This study investigated how RNA biomarkers could be effectively combined with key demographic variables to differentiate MCI or AD, predict progression from MCI to AD, and assess therapeutic effects by comparing gene expression profiles. The findings of this study may enhance the accuracy of blood-based AD diagnostic tests, enabling the more precise identification of MCI and monitoring the efficacy of disease-modifying therapies. Timely detection of MCI progression to AD is crucial, as early interventions—such as lifestyle modifications, medication, and cognitive training—could potentially delay the onset or slow the progression to AD. For instance, a two-year delay in the onset of AD could reduce the global prevalence by approximately 22.8 million cases by 2050 [33,34], highlighting the significant impact that even a modest delay in disease onset could have on the global burden of AD [35].

Our GSEA identified over one hundred DEGs that distinguish MCI or AD from CU individuals. Notably, GO terms related to T-cell, lymphocyte, and leukocyte proliferation or differentiation were significantly enriched in the CU vs. MCI comparison. Key DEGs, including *ANXA1*, *ERBB2*, *GLI3*, *SMAD7*, *CTNNB1*, *EGR3*, *PRDM1*, *SMARCD1*, and *ZFP36L2*, were significantly associated with these immunological terms. This enrichment suggests a link between early cognitive impairment and immunosenescence, aligning with the hypothesis that immune system alterations precede and potentially contribute to the early stages of the AD continuum. These findings highlight the importance of immune system dynamics in cognitive health. There is substantial evidence that immune dysregulation in AD affects both central and peripheral immune responses [36]. Given that advanced age is a primary risk factor for AD, age-related immune system changes, termed immunosenescence, are crucial to consider [37,38]. Immunosenescence involves a decline in immune function, partially explaining the increased susceptibility of older adults to infections and malignancies. Additionally, “inflammaging,” characterized by elevated levels of circulating pro-inflammatory molecules such as IL-15, contributes to age-related immune changes [39]. Further research is needed to elucidate the specific mechanisms by which immunosenescence and immune dysregulation contribute to cognitive decline. Emerging evidence indicates that the peripheral immune response, particularly involving the NF-κB signaling pathway, plays a significant role in the prodromal AD [36,40]. This association underscores the importance of systemic inflammation in the early pathogenesis of AD, highlighting the peripheral NF-κB pathway as a potential contributor to disease progression and a promising target for therapeutic intervention [41,42,43].

Our GSEA results also revealed that DEGs from the CU vs. MCI comparison were enriched for GO terms associated with the regulation of blood vessel or vascular epithelial proliferation/differentiation. Notably, the gene expression levels of consensus DEGs, including *JAG1*, *NF1*, *ACVRL1*, *FGF2*, *HIF1A*, *LEP*, *PTGS2*, *TGFBR1*, and *YAP1*, significantly discriminated MCI from CU individuals. This suggests a critical link between MCI and vascular-related processes, such as vascular dysfunction and hypoxia. Vascular abnormalities are a predominant cause of clinical dementia in the elderly. Early and persistent changes in cerebral blood flow are prominent in AD, raising the possibility of a direct relationship between vascular dysfunctions and AD pathobiology. Transcripts involved in these vascular processes have been implicated in the pathobiology of both MCI and AD. For instance, neurofibrillary tau tangles, independent of Aβ deposition, have been shown to significantly affect cortical microvasculature. These tangles promote the proliferation of small-diameter blood vessels and increase the expression of hypoxia and angiogenesis-related genes in vascular epithelial cells [44]. Numerous studies suggest that hypoxia affects many pathological aspects of AD, including oxidative stress, reactive oxygen species (ROS), and neuroinflammation, all of which have demonstrated multifaceted impacts on AD pathogenesis. Hypoxia can induce oxidative stress and disrupt cellular energy metabolism, leading to neuronal damage and dysfunction [45]. Additionally, hypoxia has been shown to promote the accumulation of misfolded Aβ and tau [46]. Furthermore, hypoxia can induce chronic inflammation and impair neurovascular function, exacerbating neurodegenerative processes [47]. These findings highlight the significance of vascular-related processes in cognitive health. However, further research is warranted to elucidate the specific mechanisms by which vascular dysfunction and hypoxia contribute to cognitive decline and to explore potential therapeutic targets for mitigating the onset and progression of neurodegenerative diseases within the vascular system.

Using GSEA and protein interaction network analysis, we also identified DEGs that distinguish AD from MCI. Notably, these DEGs were enriched for GO terms related to the regulation of exocytosis, synaptic vesicle exocytosis, vesicular trafficking, and the regulation of the secretory pathway. This enrichment underscores the importance of synaptic vesicle exocytosis and the secretory pathway in the diagnosis of AD. GO terms associated with metabolism (energy, cholesterol, and lipid) and transport pathways were also enriched. Aβ species are known to drive synaptic pathology throughout the AD continuum. Low concentrations of monomeric Aβ have been suggested to stimulate neurotransmission by enhancing vesicle docking and inhibiting neurotransmitter removal from the synaptic cleft. However, the pathological accumulation of Aβ, resulting from an imbalance between its production and clearance, predominantly blocks exocytosis, which can lead to neuronal degeneration [48,49]. Several proteins involved in extracellular vesicle functions were found to have increased levels in the AD brain. Musunuri et al. hypothesized that these changes may result in disturbed cellular clearance and perturbed cell-to-cell communication, contributing to neuronal dysfunction and cell death in AD [50]. Molecular changes leading to Aβ deposition have focused on the roles of the secretory pathway, which is crucial for the processing, quality control, and trafficking of key components of amyloidogenesis. The secretory support of amyloid precursor protein (APP) by β- and γ-secretases is a critical process involved in Aβ production [51]. Kuo et al. demonstrated that the genes contributing most to the APP support network within the secretory pathway were significantly enriched for targets of AD risk genes, suggesting a mechanistic link between genetic variants associated with AD and dysregulation of the secretory pathway [52]. These findings highlight the critical role of the secretory pathway in the pathogenesis of AD, particularly concerning Aβ production and accumulation. Overall, our results suggest that the dysregulation of exocytosis and the secretory pathway plays a significant role in the progression from MCI to AD. This finding may reflect the intricate interplay between synaptic function, vesicular trafficking, and neurodegenerative processes, emphasizing the need for a comprehensive approach to understanding and addressing the molecular underpinnings of AD. Future research should focus on elucidating the specific mechanisms by which disruptions in exocytosis and the secretory pathway contribute to AD pathogenesis.

Our analysis identified several shared biological processes that were enriched in both the MCI vs. CU and AD vs. MCI comparisons. Specifically, these included metabolic processes, such as “cellular response to ROS” and “negative regulation of TOR signaling.” These processes may reflect cellular metabolic stress and adaptation, which are central to the progression from early cognitive impairment to advanced AD. In addition, both comparisons revealed enriched GO BP terms related to cellular and biological responses, such as “regulation of intracellular signal transduction”, which may highlight the role of dysregulated intracellular signaling pathways in the pathogenesis of AD. Furthermore, we found that GO BP terms negatively associated with MCI-specific and AD-specific RNA biomarkers were linked to biological processes involving DNA repair and damage response. This shared involvement in DNA repair mechanisms suggests a common vulnerability in maintaining genomic stability during the progression from CU to MCI and subsequently from MCI to AD.

The linear mixed-effects model (LMM) incorporates both fixed and random effects, making it suitable for analyzing longitudinal repeated measures [29,53]. We employed LMM to assess the significant associations of baseline plasma proteins pTau181 and NFL with longitudinal decline in neuropsychological measures (MMSE, ADNI-MEM, ADNI-EF, ADNI-LAN, and ADNI-VS). After adjusting for potential confounding factors, including age, gender, APOE ε4, and years of education, our analysis revealed distinct differences between pTau181 and NFL. Specifically, LMM analysis indicated that individuals in the highest tertile of pTau181 (≥19.03 pg/mL) demonstrated a significant decline across all neuropsychological measures. Conversely, the highest tertile of NFL (≥37.9 pg/mL) showed significant interaction with time for all cognitive measures, except for ADNI-VS. The significant interaction effects with time suggest that elevated baseline levels of both pTau181 and NFL are associated with steeper cognitive decline.

One of the limitations of this study is the lack of external validation. To establish the clinical utility of our findings, the validation of this framework in a population-based clinical trial is essential. A large-scale study would facilitate further validation of the diagnostic and predictive performance for MCI and MCI-to-AD conversion, respectively. Moreover, this validation would provide insights into population-level analyses of MCI and AD patients and the effectiveness of AD drugs in real-world settings. Additionally, large community-based studies could help establish normal reference ranges and identify comorbidities and confounders, and adjusting for their effects could reduce disparities. Another limitation is the use of a retrospective dataset. To determine the effectiveness of ML in a clinical setting, these biomarkers should be validated using prospective clinical data.

## 5. Conclusions

This study demonstrates that machine learning-assisted analysis incorporating key demographic variables and hub RNA profiles can effectively distinguish MCI patients from CU individuals and predict the conversion of MCI to AD longitudinally. These findings suggest that these features could serve as an effective triage tool for predicting MCI-to-AD conversion, thereby optimizing therapeutic efficacy in subsequent anti-Aβ antibody treatments. Overall, this study provides a transcriptomics-based ML model and a valuable pre-screening strategy for identifying MCI patients who are ideal candidates for anti-Aβ therapy.

## Figures and Tables

**Table 1:** Baseline characteristics stratified by “diagnosis”.

	CU	MCI (Non-Converters)	MCI (AD Converters)	*p*-Value *
	(n = 78)	(n = 211)	(n = 60)	
Age	72.6 [68.0; 77.9]	69.9 [64.8; 75.6]	72.3 [68.3; 76.5]	0.007
Gender				0.406
- Female	41 (52.6%)	96 (45.5%)	25 (41.7%)	
- Male	37 (47.4%)	115 (54.5%)	35 (58.3%)	
Edu. Years	16.0 [15.0; 18.0]	17.0 [14.0; 18.0]	16.0 [14.5; 18.0]	0.315
ApoE ε4				<0.001
- 0	59 (75.6%)	126 (59.7%)	18 (30.0%)	
- 1	18 (23.1%)	70 (33.2%)	28 (46.7%)	
- 2	1 (1.3%)	15 (7.1%)	14 (23.3%)	
pTau181 (pg/mL)	13.1 [9.4; 19.0]	13.6 [9.2; 19.1]	22.1 [15.3; 28.5]	<0.001
NFL (pg/mL)	30.9 [24.4; 40.4]	30.7 [24.0; 39.8]	39.8 [28.9; 53.7]	0.001
MMSE	29.0 [29.0; 30.0]	29.0 [28.0; 30.0]	27.0 [26.0; 29.0]	<0.001
MEM	1.2 [0.7; 1.5]	0.5 [0.1; 1.0]	−0.2 [−0.6; 0.1]	<0.001
EF	0.9 [0.4; 1.6]	0.7 [0.0; 1.2]	−0.0 [−0.6; 0.5]	<0.001
LAN	1.1 [0.6; 1.4]	0.6 [0.1; 1.0]	0.1 [−0.3; 0.5]	<0.001
VS	0.7 [−0.1; 0.7]	−0.1 [−0.1; 0.7]	−0.1 [−0.8; 0.7]	0.003

* Pearson’s Chi-squared test for “gender” and “ApoE”; one-way ANOVA for the rest of variables. Values represent either the median [interquartile range] or number (% of total). Abbreviations: CU (cognitively unimpaired), MCI (mild cognitive impairment), AD (Alzheimer’s disease), pTau181 (plasma tau phosphorylated at threonine 181), NFL (neurofilament light chain), MMSE (Mini-Mental State Exam), MEM (memory), EF (executive functioning). LAN (language), and VS (visuospatial functioning).

**Table 2:** Baseline characteristics stratified by “conversion”.

	Non-Converters *	Converters	Total	*p*-Value **
	(n = 287)	(n = 62)	(n = 349)	
Age	70.8 [65.8; 76.2]	72.3 [68.3; 76.6]	71.2 [66.0; 76.2]	0.290
Gender				0.719
- Female	135 (47.5%)	27 (43.5%)	162 (46.4%)	
- Male	152 (53.0%)	35 (56.5%)	187 (53.6%)	
Edu. Years	17.0 [14.5; 18.0]	16.0 [14.0; 18.0]	16.0 [15.0; 18.0]	0.107
ApoE ε4				<0.001
- 0	184 (64.1%)	19 (30.6%)	203 (58.2%)	
- 1	87 (30.3%)	29 (46.8%)	116 (33.2%)	
- 2	16 (5.6%)	14 (22.6%)	30 (8.6%)	
pTau181 (pg/mL)	13.5 [9.4; 19.1]	21.7 [14.9; 28.4]	14.1 [10.2; 21.3]	<0.001
NFL (pg/mL)	30.7 [24.0; 40.4]	39.8 [28.0; 54.9]	31.7 [24.7; 42.1]	<0.001
MMSE	29.0 [28.0; 30.0]	27.0 [26.0; 29.0]	29.0 [27.0; 30.0]	<0.001
MEM	0.7 [0.3; 1.2]	−0.2 [−0.6; 0.1]	0.5 [0.1; 1.1]	<0.001
EF	0.7 [0.1; 1.3]	0.0 [−0.6; 0.6]	0.6 [−0.0; 1.2]	<0.001
LAN	0.7 [0.1; 1.2]	0.2 [−0.3; 0.5]	0.6 [0.0; 1.1]	<0.001
VS	0.3 [−0.1; 0.7]	−0.1 [−0.8; 0.7]	−0.1 [−0.3; 0.7]	0.003

* Non-converters comprise stable CU and stable MCI subjects. ** Pearson’s Chi-squared test for “gender” and “ApoE”; one-way ANOVA for the rest of variables. Values represent either the median [interquartile range] or number (% of total). Abbreviations: pTau181 (plasma tau phosphorylated at threonine 181), NFL (neurofilament light chain), MMSE (Mini-Mental State Exam), MEM (memory), EF (executive functioning). LAN (language), and VS (visuospatial functioning).

**Table 3:** Accuracy metrics across different AD continuum status combinations.

		CU_MCI		CU_MCI_AD		CUMCI_AD		MCI_AD	
Cut-off *p*-value		0.01	0.05	0.01	0.05	0.01	0.05	0.01	0.05
No. of probes		213	1299	223	1325	402	2227	350	1957
CU vs. MCI	Lasso	0.91	0.89	0.89	0.88	0.81	0.81	0.81	0.81
	RF	0.85	0.83	0.85	0.83	0.81	0.81	0.81	0.80
	Ridge	0.93	0.97	0.96	0.97	0.65	0.69	0.66	0.73
	SVM	0.94	0.97	0.97	0.97	0.81	0.81	0.81	0.81
MCI vs. AD	Lasso	0.15	0.2	0.3	0.21	0.63	0.47	0.54	0.44
	RF	0.00	0.00	0.00	0.00	0.21	0.04	0.24	0.05
	Ridge	0.34	0.26	0.42	0.24	0.82	0.79	0.79	0.84
	SVM	0.34	0.3	0.32	0.25	0.74	0.76	0.74	0.82

This basic model represents the accuracy metrics derived from RNA datasets alone. The heatmap color visually represent the accuracy performance of each ML algorithm, categorized by RNA probe selection conditions.

**Table 4:** Representative enriched GO terms for DEGs discriminating MCI from CU patients.

GO Description	Size	ES	NES	NOM *p*-Val
GOBP_Cellular response to reactive oxygen species	9	0.633	2.050	0.008
GOBP_Response to peptide hormone	19	0.470	2.050	0.002
GOBP_Regulation of defense response	34	0.379	2.014	0.004
GOBP_Positive regulation of canonical NF-κB signal transduction	12	0.554	1.966	0.004
GOBP_Regulation of leukocyte differentiation	21	0.428	1.956	0.006
GOBP_Cytokine production	42	0.342	1.946	0.002
GOBP_Positive regulation of immune system process	49	0.318	1.894	0.002
GOBP_Positive regulation of hemopoiesis	12	0.516	1.878	0.004
GOBP_Cell activation	49	0.322	1.846	0.002
GOBP_Regulation of intracellular signal transduction	82	0.271	1.807	0.006
GOBP_Positive regulation of intracellular signal transduction	53	0.298	1.789	0.008
GOBP_Response to insulin	12	−0.503	−1.807	0.002
GOBP_Fat cell differentiation	16	−0.465	−1.866	0.008
GOBP_Regulation of DNA metabolic process	24	−0.456	−2.096	0.002
GOBP_Double-strand break repair	14	−0.571	−2.181	0.002

Abbreviations: ES, enrichment score; NES, normalized enrichment score; NOM *p*-val, nominal *p*-value.

**Table 5:** Representative enriched GO terms for DEGs discriminating AD from MCI.

GO Description	Size	ES	NES	NOM *p*-Val
GOBP_Regulated exocytosis	23	0.521	2.230	0.000
GOBP_Neurotransmitter secretion	14	0.605	2.207	0.000
GOBP_Regulation of membrane repolarization	6	0.810	2.105	0.000
GOBP_TOR_signaling	11	0.620	2.081	0.002
GOBP_Exocytosis	37	0.397	1.989	0.004
GOBP_Positive regulation of intracellular protein transport	11	0.593	1.984	0.002
GOBP_Regulation of regulated secretory pathway	11	0.600	1.951	0.000
GOBP_Adipose tissue development	9	0.623	1.943	0.002
GOBP_Positive regulation of ROS species metabolic process	8	0.652	1.918	0.004
GOBP_Negative regulation of TOR signaling	8	0.650	1.882	0.004
GOBP_Cellular response to cAMP	6	0.732	1.876	0.004
GOBP_Vesicle docking	8	0.648	1.870	0.006
GOBP_Cellular modified amino acid metabolic process	15	0.486	1.834	0.010
GOBP_Export from cell	94	0.268	1.735	0.002
GOBP_Microtubule cytoskeleton organization	49	−0.332	−1.758	0.009
GOBP_DNA metabolic process	70	−0.329	−1.905	0.000
GOBP_DNA damage response	57	−0.361	−1.978	0.004
GOBP_Negative regulation of cell adhesion	26	−0.457	−2.006	0.000
GOBP_Organelle fission	29	−0.462	−2.049	0.000
GOBP_DNA repair	31	−0.480	−2.213	0.000

Abbreviations: ES, enrichment score; NES, normalized enrichment score; NOM *p*-val, nominal *p*-value.

**Table 6:** Top 30 RNA biomarkers with the highest hazard ratios (HR) for predicting MCI-to-AD conversion.

Gene	Probe	β	SE	z	*p* Value	HR (95% CI)
GPD1	11720473_at	1.025	0.121	8.452	2.86 × 10^−17^	2.79 (2.20–3.54)
HAP1	11731552_a_at	0.922	0.110	8.407	4.20 × 10^−17^	2.51 (2.03–3.12)
ITGAM	11732481_a_at	0.864	0.119	7.277	3.42 × 10^−13^	2.37 (1.88–2.99)
CBS	11744287_x_at	0.776	0.110	7.080	1.44 × 10^−12^	2.17 (1.75–2.69)
DIP2B	11717068_a_at	0.728	0.105	6.924	4.39 × 10^−12^	2.07 (1.69–2.54)
HRH2	11740951_s_at	0.723	0.109	6.634	3.27 × 10^−11^	2.06 (1.66–2.55)
LILRB3	11745488_s_at	0.706	0.107	6.610	3.84 × 10^−11^	2.03 (1.64–2.50)
GPR68	11724423_a_at	0.642	0.109	5.872	4.31 × 10^−9^	1.90 (1.53–2.36)
FBXL20	11729398_a_at	0.638	0.103	6.170	6.82 × 10^−10^	1.89 (1.55–2.32)
SLC12A1	11728244_s_at	0.634	0.105	6.007	1.89 × 10^−9^	1.88 (1.53–2.32)
NPPA	11757468_a_at	0.623	0.105	5.930	3.03 × 10^−9^	1.86 (1.52–2.29)
TLR6	11737628_a_at	0.621	0.108	5.775	7.71 × 10^−9^	1.86 (1.51–2.30)
CBS	11744835_s_at	0.618	0.101	6.090	1.13 × 10^−9^	1.85 (1.52–2.26)
SLC12A1	11752597_a_at	0.615	0.107	5.761	8.37 × 10^−9^	1.85 (1.50–2.28)
KCNB1	11732588_at	0.591	0.111	5.317	1.06 × 10^−7^	1.81 (1.45–2.25)
CYP4F2	11727964_x_at	0.591	0.109	5.428	5.71 × 10^−8^	1.81 (1.46–2.24)
RAB11FIP1	11761457_at	0.590	0.101	5.868	4.40 × 10^−9^	1.80 (1.48–2.20)
DIO1	11729362_a_at	0.575	0.103	5.591	2.25 × 10^−8^	1.78 (1.45–2.17)
SPTBN4	11734303_a_at	0.574	0.106	5.432	5.57 × 10^−8^	1.78 (1.44–2.18)
CBS	11744286_s_at	0.569	0.101	5.619	1.92 × 10^−8^	1.77 (1.45–2.15)
CSGALNACT1	11732525_a_at	0.563	0.105	5.335	9.55 × 10^−8^	1.76 (1.43–2.16)
MTM1	11749427_a_at	0.561	0.109	5.161	2.45 × 10^−7^	1.75 (1.42–2.17)
ACVRL1	11747260_a_at	0.537	0.105	5.133	2.86 × 10^−7^	1.71 (1.39–2.10)
RNF152	11732769_at	0.537	0.102	5.279	1.30 × 10^−7^	1.71 (1.40–2.09)
ADIPOQ	11734559_x_at	0.522	0.101	5.178	2.24 × 10^−7^	1.69 (1.38–2.05)
CAV1	11757013_x_at	0.525	0.108	4.864	1.15 × 10^−6^	1.69 (1.37–2.09)
DPYSL5	11739423_at	0.518	0.109	4.759	1.95 × 10^−6^	1.68 (1.36–2.08)
PPY	11730869_s_at	0.512	0.105	4.888	1.02 × 10^−6^	1.67 (1.36–2.05)
CHDH	11739355_at	0.514	0.104	4.921	8.62 × 10^−7^	1.67 (1.36–2.05)
WDR1	11745608_a_at	0.500	0.108	4.641	3.47 × 10^−6^	1.65 (1.34–2.04)

Abbreviations: β, the regression coefficient; SE, standard error of β; z, Wald statistic value (z = β/SE); HR (95% CI), hazard ratio (95% confidence interval).

**Table 7:** Association of plasma biomarkers with neuropsychological scores.

Cognition Measure	Predictors	β	SE	*t*	*p* Value
MMSE	pTau181 × time	−0.381	0.059	−6.513	<0.001
	pTau181	0.040	0.261	0.153	0.879
	NFL × time	−0.285	0.059	−4.828	<0.001
	NFL	0.554	0.263	2.111	0.036
ADNI-MEM	pTau181 × time	−0.067	0.010	−6.503	<0.001
	pTau181	−0.156	0.088	−1.764	0.080
	NFL × time	−0.061	0.010	−5.875	<0.001
	NFL	0.057	0.087	0.659	0.511
ADNI-EF	pTau181 × time	−0.058	0.013	−4.516	<0.001
	pTau181	−0.052	0.098	−0.529	0.597
	NFL × time	−0.067	0.013	−5.270	<0.001
	NFL	0.082	0.095	0.862	0.390
ADNI-LAN	pTau181 × time	−0.060	0.013	−4.625	<0.001
	pTau181	−0.034	0.084	−0.408	0.684
	NFL × time	−0.055	0.013	−4.209	<0.001
	NFL	0.148	0.082	1.805	0.073
ADNI-VS	pTau181 × time	−0.029	0.015	−2.007	0.045
	pTau181	−0.022	0.069	−0.319	0.750
	NFL × time	−0.017	0.014	−1.193	0.233
	NFL	0.037	0.068	0.546	0.586

Abbreviations: β, the regression coefficient; SE, standard error of β; *t*, T-statistic value (*t* = β/SE). The LMM was adjusted for age, sex, ApoE ε4 alleles, and years of education. Protein × time denotes the time interaction effect of the protein.

## Data Availability

The data presented in this study are available on request to the corresponding author.

## Supplementary Materials

The following supporting information can be downloaded at: https://www.mdpi.com/article/10.3390/cells13221920/s1, Figure S1: Associations of demographics and neuropsychological measures with disease status classification. Figure S2: Correlations heatmap on MCI probes. Figure S3: LMM plots for ADNI-EF and ADNI-LAN. Table S1: RNA biomarkers predicting for MCI-to-AD conversion.
