# Supplementary material for: A Transcriptomics-Based Machine Learning Model Discriminating Mild Cognitive Impairment and the Prediction of Conversion to Alzheimer’s Disease
Source: Cells. 2024 Nov 19;13(22):1920. doi: 10.3390/cells13221920 (PMC11593234; doi:10.3390/cells13221920)
Supplement: Supplementary file 1 [file cells-13-01920-s001.zip › Fig._S3.docx]

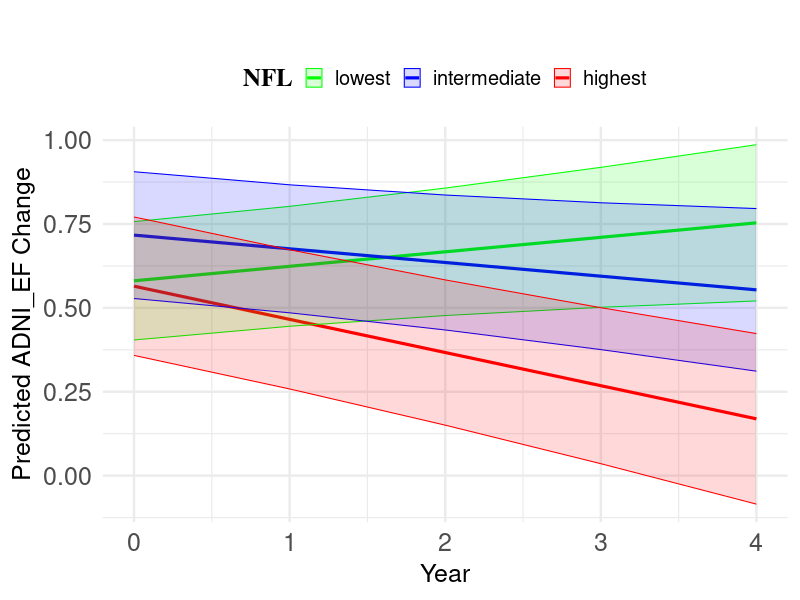

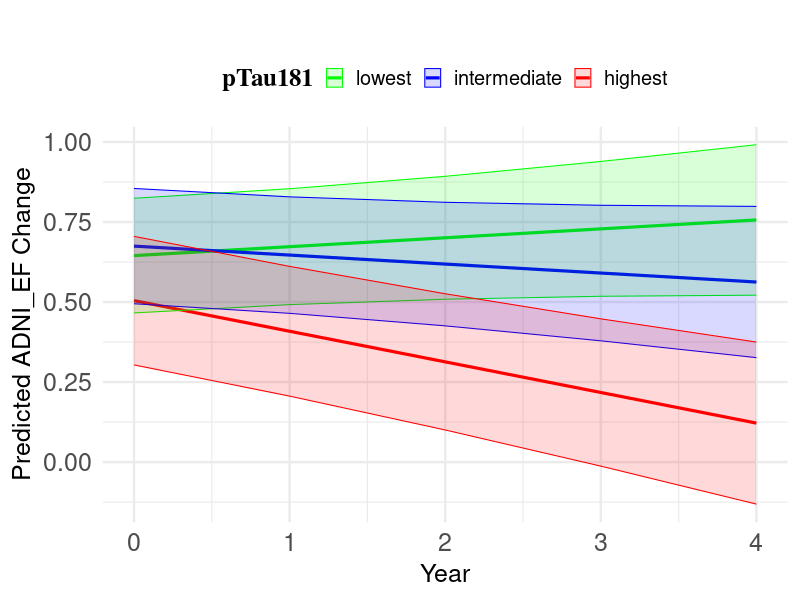
(a) (b)


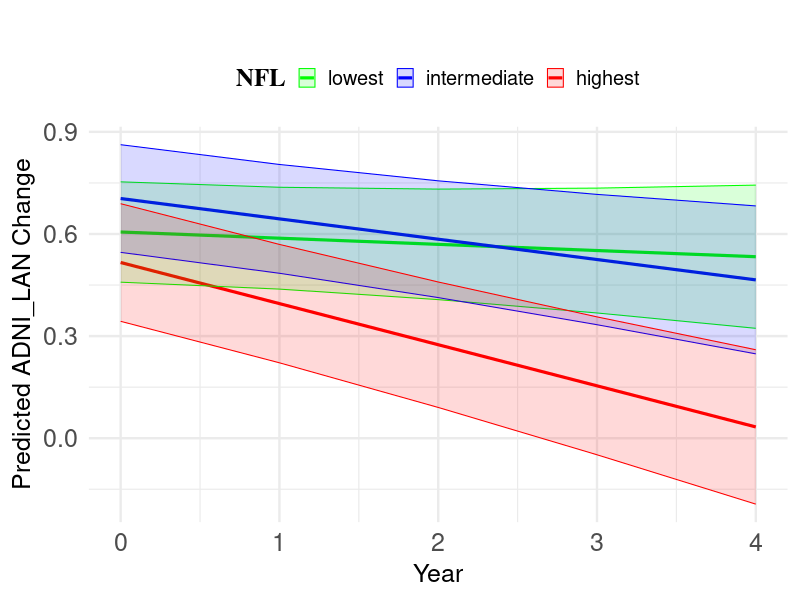

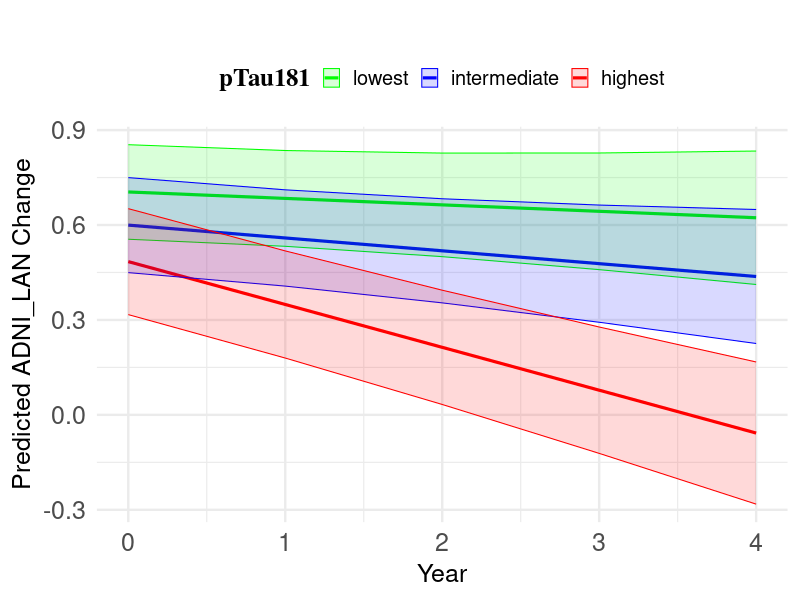
(c) (d)


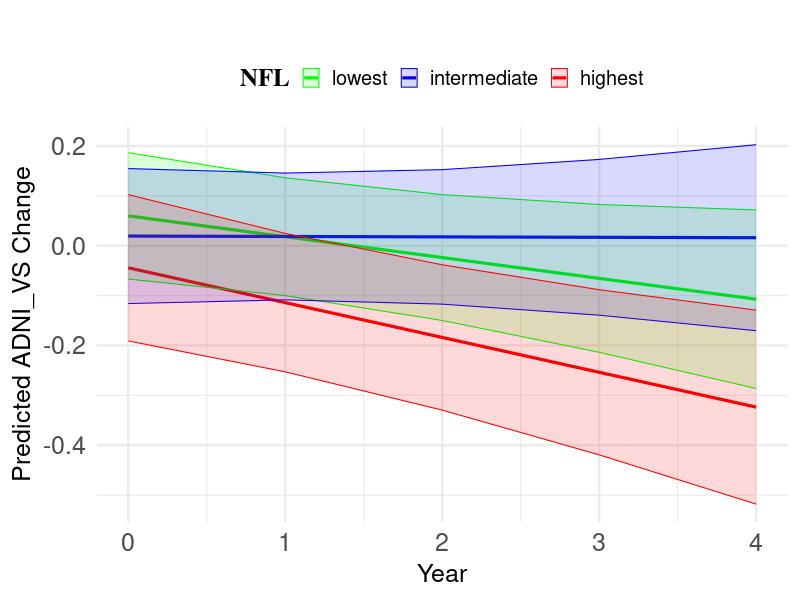

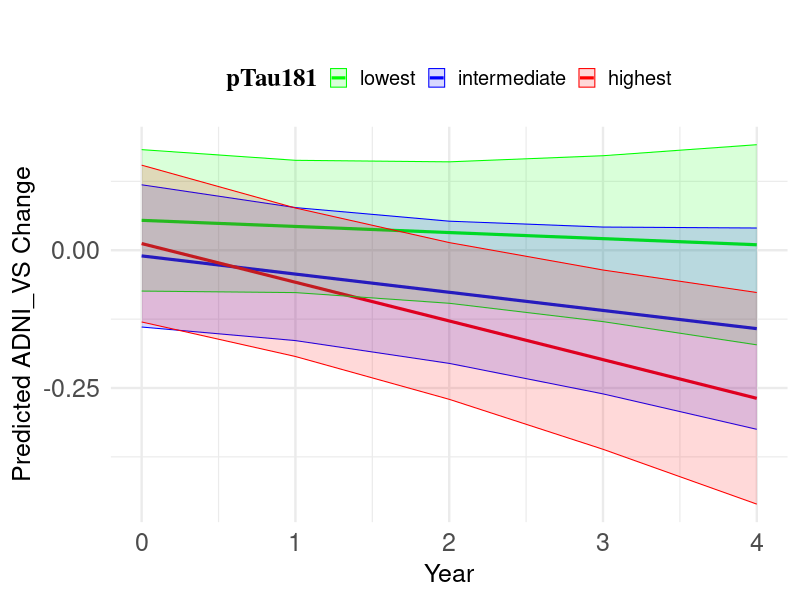
(e) (f)

**Figure S3.** Prediction of baseline proteins on longitudinal neuropsychological score alterations. Trajectories were derived from LMM with baseline plasma pTau181 and NFL levels as predictors, adjusted for age, sex, ApoE ε4, and years of education. ADNI-EF trajectories stratified by (a) pTau181 or (b) NFL tertiles. ADNI-LAN trajectories stratified by (c) pTau181 or (d) NFL tertiles. VisADNI trajectories stratified by (e) pTau181 or (f) NFL tertiles. The trajectories depict alterations in ADNI-MEM, -LAN, or -VS scores over time influenced by varying tertiles of baseline pTau181 or NFL levels. The slope, indicative of the rate of cognitive decline, appears steeper for individuals with elevated protein levels. The red line represents the highest tertile of each protein, while the blue and green lines represent the intermediate and lowest tertiles, respectively. Shaded areas indicate the 95% confidence intervals of the regression lines. This figure depicts mean levels within each covariate (age and years of education), with females as the reference group. The time span was capped at four years, corresponding to four follow-up assessments.
